# Melting Temperature Under Pressure in ‘Hard’ and Soft Matter

**DOI:** 10.3390/ijms27177568

**Published:** 2026-08-24

**Authors:** Aleksandra Drozd-Rzoska, Sylwester J. Rzoska, Izabella Grzegory, Sylwester Porowski

**Affiliations:** Institute of High Pressure Physics of the Polish Academy of Sciences, ul. Sokołowska 29/37, 01-142 Warsaw, Poland

**Keywords:** melting temperature, high pressure, negative pressure, soft matter, molecular structure, molecular interactions

## Abstract

This report focuses on cognitive gaps of the pressure dependence of melting temperature, presenting: (*i*) a coherent discussion on $T_{m}(P)$ behavior for both ‘Hard Matter’ (‘standard’ solid state) and Soft Matter systems, to reveal the importance of the strength of intermolecular interactions; (*ii*) a model picture linking systems with $dT_{m}/dP>0$, $dT_{m}/dP<0$, and the $T_{m}\left(P\right)$ curve maximum, which also shows the relevance of the negative-pressure domain; and (*iii*) the ultimate validation test of the recent ‘universal’ parabolic scaling of $P_{m}(T)$ against empirical data. The evidence for ‘Hard Matter’ focuses on systems relevant to the semiconductor industry: silicon, germanium, and gallium nitride. For Soft Matter, these include unique polymeric systems and liquid-crystalline pentylcyanobiphenyl (5CB). For the latter, symmetry-selected melting/freezing takes place. Finally, the report presents the specific case of graphite and diamond. The report also presents an innovative solution for melting-temperature and pressure detection.

## 1. Introduction

Melting is one of the most important phenomena in physical chemistry, earth sciences, and materials engineering. It also shapes a variety of natural processes, from deep-Earth geophysics to the melting of glaciers and large ice sheets [1,2,3]. Melting also underpins numerous technological applications, from metallurgy [4,5] and the chemical industry [6,7] to pharmacy [8,9,10] and food technology and preservation [10,11,12].

Notwithstanding, melting discontinuous transitions still constitute a significant cognitive challenge for which ‘classic’ Clausius–Clapeyron (C-C, 1837/1859, [13,14]) and Lindemann (1910, [15]) model relations make up the basic reference.

The C-C equation [13,14] focuses on step changes in basic thermodynamic properties when passing the melting temperature $T_{m}$, namely,

(1) $$\frac{dT_{m}}{dP}=T_{m}\frac{\Delta V}{L}=\frac{\Delta V}{\Delta S}\;⟹\;\frac{1}{T_{m}\left(P\right)}\frac{dT_{m}\left(P\right)}{dP}=\frac{dT_{m}\left(P\right)/T_{m}\left(P\right)}{dP}=\frac{dlnT_{m}\left(P\right)}{dP}=\frac{\Delta V\left(P\right)}{\Delta H\left(P\right)}$$

where ΔV and ΔH are the volume and enthalpy step changes; the latter can be alternatively expressed as the latent heat L. The convenient metric of the transition is the entropy change: $\Delta S=L/T_{m}$.

Lindemann (1910, [15]) proposed a semi-microscopic picture by linking melting to the mean-squared displacement of particles in a crystalline solid, finally leading to fluidization. It was concluded in the following relation [15,16,17]:

(2) $$\frac{dT_{m}\left(P\right)}{dP}=2T_{m}\left(\gamma_{G}-\frac{1}{3}\right)B_{T}^{-1}\;\Rightarrow \;\frac{dT_{m}\left(P\right)/T_{m}\left(P\right)}{dP}=\frac{dlnT_{m}\left(P\right)}{dP}=2\left(\gamma_{G}-\frac{1}{3}\right)B_{T}^{-1}\;$$

where $B_{T}={\left(-VdP/dV\right)}_{{T⟶T}_{m}}$ is the bulk modulus, i.e., the metric of the system’s relative resistance to compression, and $\gamma_{G}$ means the Grüneisen parameter.

The Grüneisen parameter describes relative changes in vibrational frequencies of atoms in the crystal lattice with respect to relative volume changes [16,17]:

(3) $$\gamma_{G}=-\frac{d\omega /\omega }{dV/V}=-\frac{dln\omega }{dlnV}=-\frac{dln\theta_{D}}{dlnV}$$

where ω stands for the angular frequency and $\theta_{D}$ is for the Debye temperature.

The thermodynamic definition of the Grüneisen parameter includes the specific heat $c_{V}$, thermal expansion coefficient $\alpha_{T}$ and bulk modulus $B_{T}$: $\gamma_{G}=\alpha_{T}B_{T}V/c_{V}$ [16,17].

Both the C-C and Lindemann relations emphasize the impact of pressure on the melting temperature. This problem was taken up in 1929 by Simon and Glatzel (SG), who proposed an empirical relation—still the basic ‘tool’ for describing such changes [18]:

(4) $$P=-a\prime +b\prime T^{b}\;(\mathrm{along}\;\mathrm{melting}\;\mathrm{curve})$$

In subsequent decades, another form of Equation (4) became dominant [5,19,20,21,22,23,24,25,26,27]:

(5) $$T_{m}\left(P\right)=T_{0}{\left(1+\frac{P}{a}\right)}^{1/b}\Rightarrow (\mathrm{along}\;\mathrm{melting}\;\mathrm{curve})\;\Rightarrow P=-{a\left(T_{0}\right)}^{b}+{aT}^{b}\;$$

where $T_{0}$ is the reference pressure standard linked to the reference atmospheric pressure, and is approximated as follows: $P_{0}=0.1\;MPa\to P_{0}=0$.

Equation (5) also shows the link to the basic SG formulation given in Equation (4).

Although Simon and Glatzel [18] had already declared inspiration from the C-C relation, an unambiguous derivation of Equation (5) on this basis was reported by Boguslavskii only in 1982 [28]. Earlier, the SG was derived from the Lindemann model dependence by Gilvarry [29], who also showed the possible direct link of relevant parameters in Equation (5) to the interaction potential on the solid, crystalline side of $T_{m}$: $a=B_{0}/B^{\prime }$ and $b=\left(2\gamma -1\right)/\left(3+f\right)$, where $B_{0}$ is the bulk modulus (compressibility) under atmospheric pressure and $B^{\prime }$ is its derivative indicating pressure dependence, and *f* is the parameter related to the Morse interatomic interaction model.

The SG equation describes the permanent increase in melting temperature on compression (i.e., $dT_{m}/dP>0$), characteristic of most of the systems studied so far. However, there are also less common systems where $dT_{m}/dP<0$ or the maximum of the melting curve appears, i.e., $dT_{m}/dP>0\;\to \;dT_{m}/dP<0$ crossover on compression [19,23]. In such a case, the following relation has become the standard for empirical data scaling [22,23,24,25,26,27,30,31,32]:

(6) $$T_{m}\left(P\right)=R\left(T\right)D\left(T\right){=T}_{0}{\left(1+\frac{P}{a}\right)}^{1/b}exp\left[-c\times P\right]\;$$

where c,a,b=const and $T_{0}$ is the reference melting temperature under atmospheric pressure. R(T) represents an SG-type rising term and D(T) the exponential damping term.

The derivation of Equation (6) is based on the C-C equation, with the following leading pressure dependence, $dlnT_{m}/dP=y=y_{0}+y_{0}^{\prime }\Delta P+y_{0}^{\prime \prime }{\left(\Delta P\right)}^{2}$, where $\Delta P=P-P_{0}$.

Equation (6) is commonly referred to as the ‘Kechin equation’ [22,23,24,25,26,27,33,34,35,36,37,38,39,40,41], which may be related to the impact of papers published in 1995 and 2001 [30,31]. However, Rein and Demus [32] reported the same equation in 1992, following the same reasoning. The authors prefer to name Equation (6) as the Rein–Demus–Kechin (RDK) relation, which is used further in this report. The derivation of Equation (6) using the Lindemann model relation is given by Arafin et al., in ref. (2013, [42]).

A comprehensive and in-depth presentation of the state-of-the-art melting transition is given in topical reviews [22,23,24,25]. Particularly notable is the most recent one by de With (2023, [25]) under the symptomatic title ‘*Melting is well-known, but is it also well-understood?*’. It terminates with the pessimistic conclusion [25]: ‘ …*it may well be that a generally applicable mechanistic theory, in view of the many aspects that require attention, does not exist*’. Refs. [22,23,24,25] also contain extensive presentations of ‘classic’ approaches to the pressure dependence of the melting temperature problem (Equations (4)–(6)).

The cognitive pessimism regarding the fundamental insight stems from the fact that despite nearly two centuries of research, the Clausius–Clapeyron and Lindemann model relations, supported by thermodynamic studies and structural analysis of the solid crystalline phase side of $T_{m}$, remain the essential research reference for this topic [19,20,21,22,23,24,25,26,27,28,29,30,31,32,33,34,35,36,37,38,39,40,41,42].

Above, only a brief summary of the current state of the art on discontinuous melting transitions and the impact of pressure is presented, focusing on issues relevant to the results of this report presented below.

This cognitive picture should be supplemented by the very recent milestone report by Trachenko (2024, [43]), who derived the hypothetically universal relation for any material equation:

(7) $$P_{m}\left(T\right)=D_{1}+D_{2}T+D_{2}T^{2}\;(\mathrm{along}\;\mathrm{the}\;\mathrm{melting}\;\mathrm{line})$$

with fundamentally well-defined parameters $D_{i}=const$.

What is particularly noteworthy is that it was derived from the Phonon Theory of Liquids [43], discussed beyond the ‘classic’ pattern recalled above. This work immediately aroused enormous [44,45,46,47,48,49] and still-lasting interest; in the first 7 months of 2026, it is recalled in at least 11 reports [50,51,52,53,54,55,56,57,58,59,60].

This report also discusses issues related to Trachenko’s scaling [43] in the Section 3, where the essential and still-unresolved problem of its ultimate empirical validation is finally addressed.

**Figure 1:**
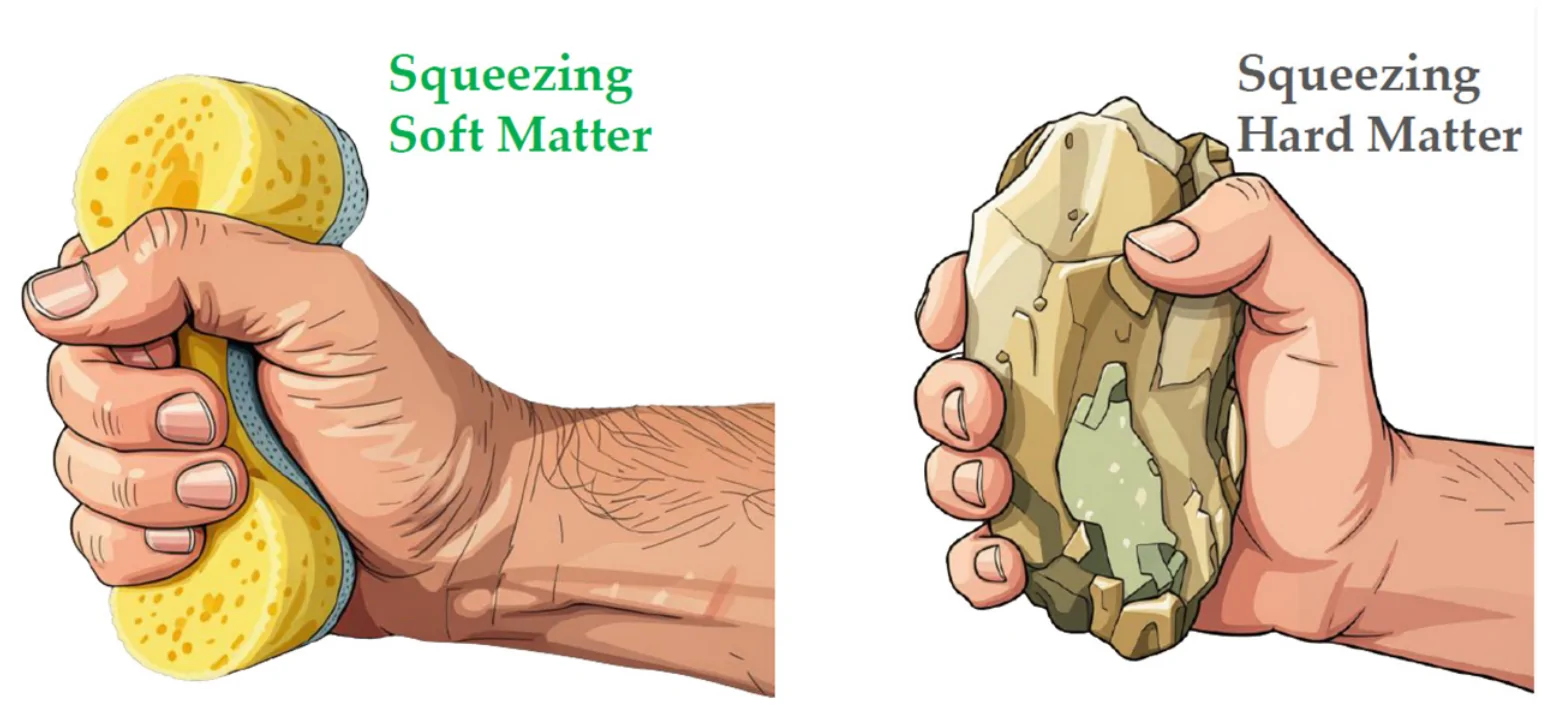
An illustration of the impact of pressure on a Sponge (Soft Matter) and a Stone (Hard Matter, standard solid state). The hand of S.J. Rzoska (the co-author).

However, a significant cognitive gap remains in the review reports published so far [22,23,24,25,26,27]: they completely omit so-called Soft Matter systems [61,62,63,64,65,66]. This category, introduced by Pierre G. de Gennes in his Nobel Prize Lecture (1991) [61,62,63], links liquid crystals, polymers, colloids, and biosystems, among others. Despite these qualitative differences, they exhibit universalistic features resulting from the dominance of weak intermolecular interactions and mesoscale spaces, which also leads to very high sensitivity to exogenous impacts, including pressure [64]. The essential difference in the impact of pressure on standard solid-state materials (‘Hard Matter’) and Soft Matter is illustrated in Figure 1. For Soft Matter systems, (*i*) they are governed by mesoscale forming units, from macromolecules to multimolecular assemblies, with weak interaction energy $\sim kT_{B}$ or even lower; (*ii*) the response to exogenous impacts (temperature, pressure, electric field) is adaptive or elastic.

For standard or ‘Hard Matter’ systems, (*i*) they are associated with atomic-scale forming units, with covalent, ionic, or metallic bondings related to large energy $>>kT_{B}$; (*ii*) they are ‘rigid’ or near-fixed to even relatively strong impacts of external fields.

Generally, intermolecular or between-multimolecular assembly interactions are 100×–1000× or even ‘weaker’ in Soft Matter systems than in standard ‘Hard Matter’ ones. Notably, in Soft Matter systems, melting can occur even for a single, specific element of symmetry, which is definitely not the case for ‘Hard Matter’ [64].

This report focuses on cognitive gaps regarding the pressure dependence of the melting temperature considering both ‘Hard Matter’ and Soft Matter systems, namely:

- A coherent discussion of $T_{m}(P)$ behavior both for ‘Hard Matter’ (‘standard’ solid state) and Soft Matter systems, which can reveal the significance of the strength of intermolecular interactions.
- A model description enabling a coherent picture of systems characterized by $dT_{m}/dP>0$, $dT_{m}/dP<0$, and the $T_{m}\left(P\right)$ curve maximum. It also shows the relevance of the negative-pressure domain.
- The ultimate validation of the Trachenko scaling relation against empirical data.
- The exceptional primary relaxation time invariance along the melting curve in the *P-T* plane for symmetry-selected transitions in Soft Matter systems.

The discussion of ‘Hard Matter’ systems explores $T_{m}(P)$ data for systems relevant to the semiconductor industry, but not coherently presented in review reports so far. The meaning of intermolecular and interatomic interactions and the distinction between ‘Hard Matter’ and ‘Soft Matter’ are also discussed. Finally, the paper discusses some new significant experimental issues.

## 2. Results

For solids and liquids, the value P=0 is assumed in Equations (5) and (6) as the reference. It is considered a reasonable approximation of the atmospheric pressure P=0.1 MPa for the given phenomenon. However, P=0 is not a terminal value for solids or liquids. Namely, a smooth crossover from the compressed domain (P>0) to the isotropically stretched (P<0, negative pressures) domain is possible [67]. It terminates at the absolute stability limit of spinodal pressure $P_{Sp}$ [67]. Drozd-Rzoska implemented this fact in Equation (6), and proposed the following equation [23,68]:

(8) $$T_{m}\left(P\right)=T_{ref.}{\left(1+\frac{\Delta P}{\Pi }\right)}^{1/b}exp\left(-\Delta P/\theta \right)$$

where $\Delta P=P-P_{ref.}$, $\Pi =\pi -P_{ref.}$, −*π* is the extrapolated (negative) pressure for which $T_{m}\left(P\to -\pi \right)\to 0$, and θ is the reference damping pressure, related to c=1/θ in Equation (6). $\left(T_{ref.},P_{ref.}\right)$ are the reference temperature and pressure along the melting curve. For θ ⟶ ∞ and $P_{ref.}=0$, the basic Simon–Glatzel equation is retrieved.

When comparing parameters in Equations (6) and (8), one obtains Π=a and θ=1/c. The reference pressure $P_{ref.}$ also appeared earlier, but it was linked to the triple-point coordinates in the ‘standard, positive’-pressure domain [30,31].

Usually, the nonlinear fitting of experimental data is performed using a model scaling relation over the available experimental data pressure range. However, one can reverse this protocol and first focus on whether, and to what extent, a scaling relation can optimally describe the empirical dataset. For $T_{m}\left(P\right)$ scaling, it can be realized via the following transformation of empirical data, directly resulting from Equation (8) [23,68,69]:

(9) $$T_{m}\left(P\right)\;⟶\;{\left[\frac{d\left(lnT_{m}\right)}{dP}\right]}^{-1}=b+\left(b/\pi \right)\Delta P$$

(10) $$T_{m}\left(P\right)\;⟶\;{\left[\frac{d\left(lnT_{m}\right)}{dP}+\theta^{-1}\right]}^{-1}=b+{\left(b/\pi \right)}_{eff.}\Delta P$$

Equation (9) is related to the case of the permanent rise in $T_{m}$ with compression (no ‘damping’ term), and can also be considered the Simon–Glatzel equation distortion-sensitive validation test. Equation (10) is the distortion-sensitive test for Equation (8) and also the RDK equation (Equation (6)).

In summary, Equations (9) and (10) define the ‘*bottom-up*’ protocol for $T_{m}\left(P\right)$ scaling of empirical data, with the following steps [23,68,69]:

- Empirical data are transformed first following Equation (9). A linear domain validates the description of the basic S-G equation (Equation (6)).
- If the above test does not yield a linear behavior, the supplementary test via Equation (10) is advised, with subsequent steps of π parameter values until reaching a linear behavior.
- When obtaining a linear behavior, the linear regression fit can yield all relevant parameters in Equation (8) with well-defined errors.
- The obtained parameters can be directly substituted into Equation (8), and no further fitting is needed to portray experimental data.
- Note the inherent allowance to enter the negative-pressure domain.

Equation (9) or (10) represents the ‘bottom→up’ protocol, first focusing on the preferences of empirical data.

**Figure 2:**
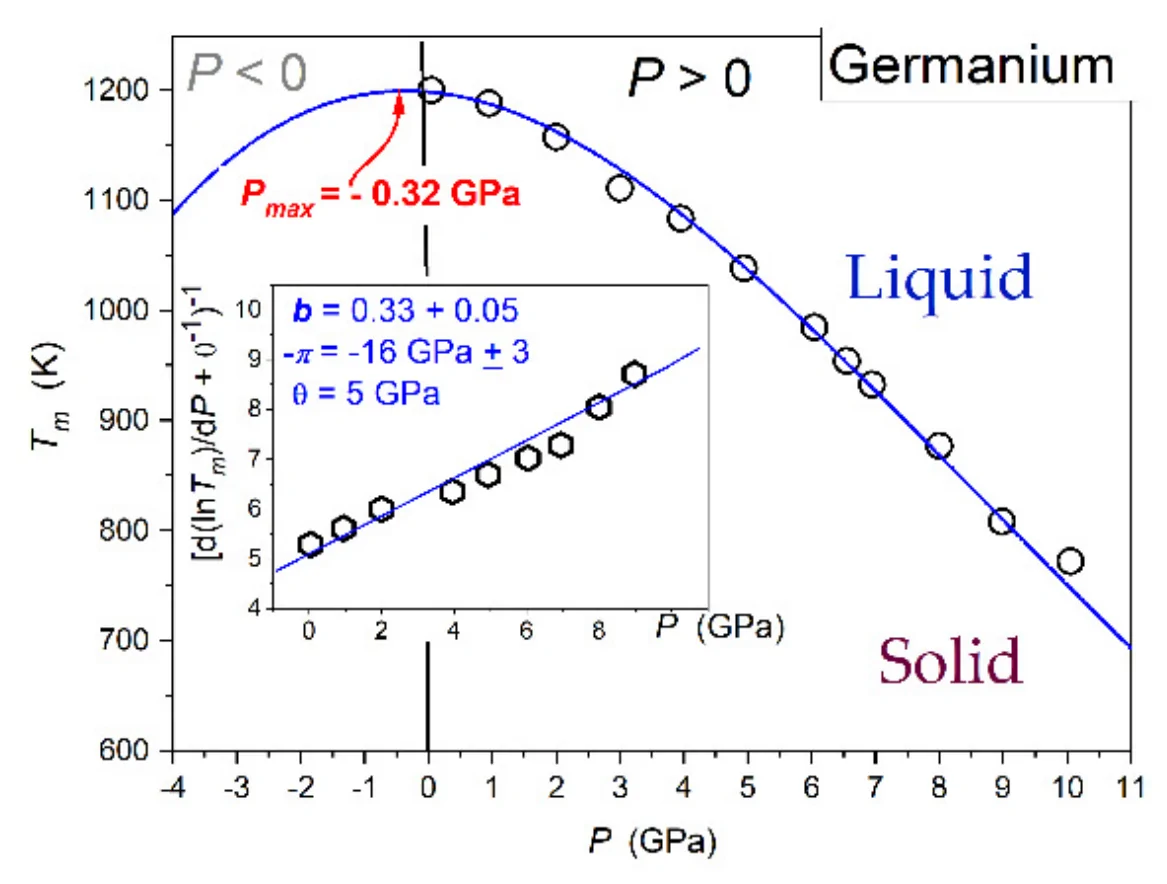
The melting curve for germanium is portrayed via Equation (8), with the supporting analysis via Equation (10), validating the description and yielding optimal parameter values. The analysis reveals the $T_{m}(P)$ curve maximum hidden in the negative-pressure domain, indicated in the plot. The plot was prepared using the authors’ data, preliminarily reported in ref. [23].

**Figure 3:**
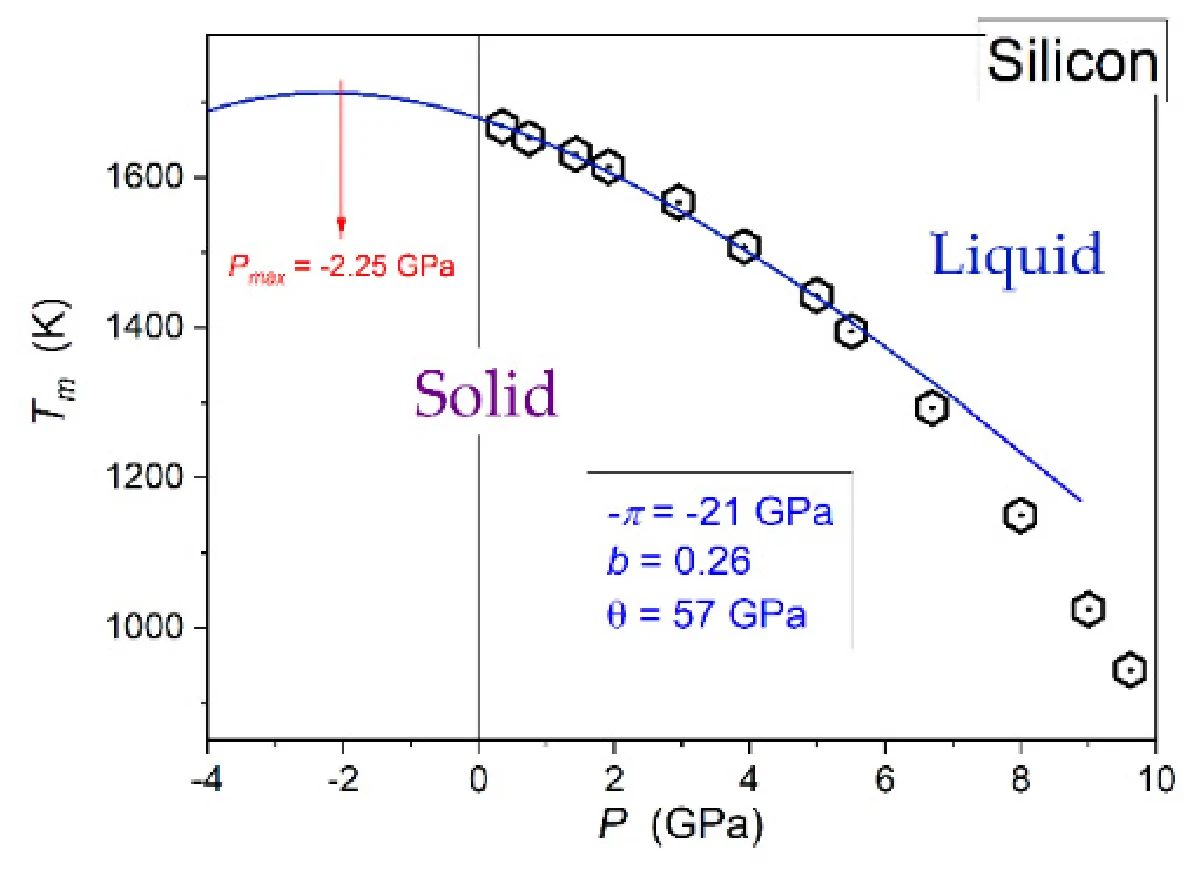
The melting curve for silicon, showing the increasing reversed melting on compression. The analysis via Equations (8) and (10) reveals the $T_{m}(P)$ curve maximum hidden under negative pressures, as indicated in the plot. The plot was prepared using the authors’ data, reported first in ref. [23].

**Figure 4:**
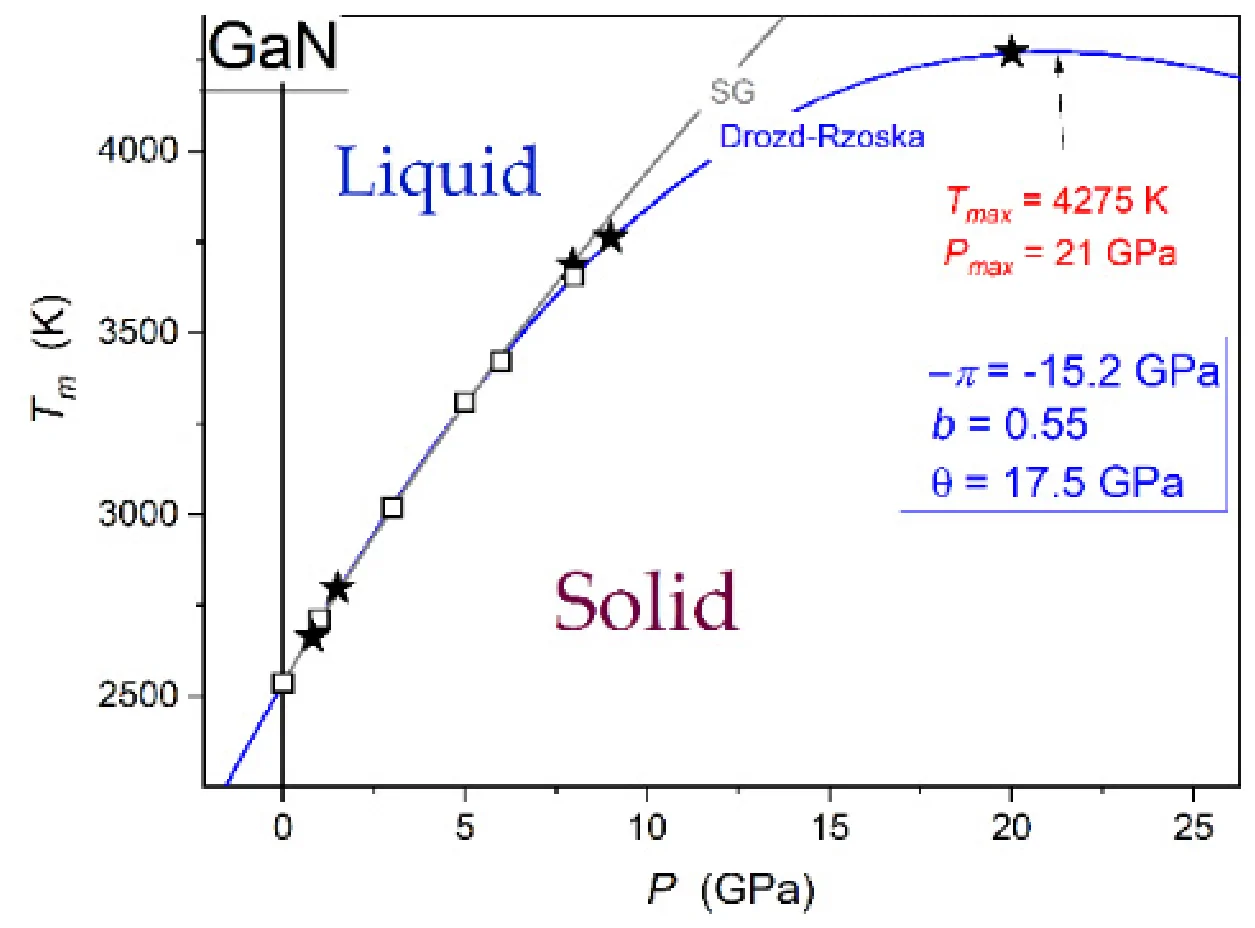
The hypothetical melting curve $T_{m}\left(P\right)$ for gallium nitride (GaN) with the portrayal via the Drozd-Rzoska equation (Equation (8)), which revealed the possible maximum. Note the extension into the negative-pressure domain. GaN decomposes before melting, but the hypothetical melting temperature can be estimated using the protocol described above and in refs. [23,68,69]. The plot suggests that a direct melting transition is possible for P>12 MPa. The plot was prepared using the authors’ data and analysis, first presented in ref. [70]. Note the extension into the negative-pressure domain.

Figure 2, Figure 3 and Figure 4 below present $T_{m}\left(P\right)$ data for basic semiconductor-relevant material, scaled by Equation (8), with the support of the above protocol and Equation (10).

They are germanium (Ge) and silicon (Si) with the inherent $dT_{m}/dP<0$ pattern, and gallium nitride (GaN) for which the melting pattern had remained puzzling until the Drozd-Rzoska equation (Equation (8)) [23,68,69] and the above protocol were implemented in ref. [70].

For silicon and germanium, the melting temperature decreases permanently with compression, i.e., $dT_{m}/dP<0$. The scaling via Equation (8), supported by the preliminary analysis using Equation (10), reveals that it can be associated with the melting curve maximum hidden in the negative-pressure domain.

Gallium nitride (GaN) is one of the most innovative materials, having already revolutionized the world by introducing new light sources far more efficient than the light bulbs that dominated for over a century. GaN is also associated with the 2014 Nobel Prize in Physics awarded jointly to Isamu Akasaki, Hiroshi Amano, and Shuji Nakamura ‘*for the invention of efficient blue light-emitting diodes which has enabled bright and energy-saving white light sources*’ [71].

It is also considered a significant base for high-power and high-frequency transistors and converters [72]. The discussion of $T_{m}(P)$ dependence for GaN had remained inconclusive for decades, despite its fundamental and practical significance [72,73,74,75,76]. For a long time, the picture was dominated by the model proposed by van Vechten [73], which heuristically suggests $dT_{m}/dP<0$. For experiments, the essential problem constituted the decomposition of GaN before melting. Finally, the problem was explained in ref. [70], where the behavior related to $dT_{m}/dP>0$ and the maximin of the $T_{m}(P)$ curve, supported by fair scaling of the Drozd-Rzoska equation (Equation (8)), was finally evidenced. The $T_{m}(P)$ maximum is expected at ~21 GPa, which is indicated in Figure 4.

The above materials can be named ‘**Hard Matter**’, for which strong inter-elemental bondings cause multi-GPa pressures to be needed to significantly change properties, including the melting temperature. The ‘**Soft Matter**’ category is associated with systems dominated by mesoscale elements, such as macromolecules or molecular assemblies, where forces associated with inter-elemental bondings are essentially weaker.

To assess the effect of pressure on a given material, it is important to compare the related forces of inter- or intramolecular interactions in a given system. Conceptually, the difference between ‘Hard Matter’ and Soft Matter under compression is shown in Figure 1.

**Figure 5:**
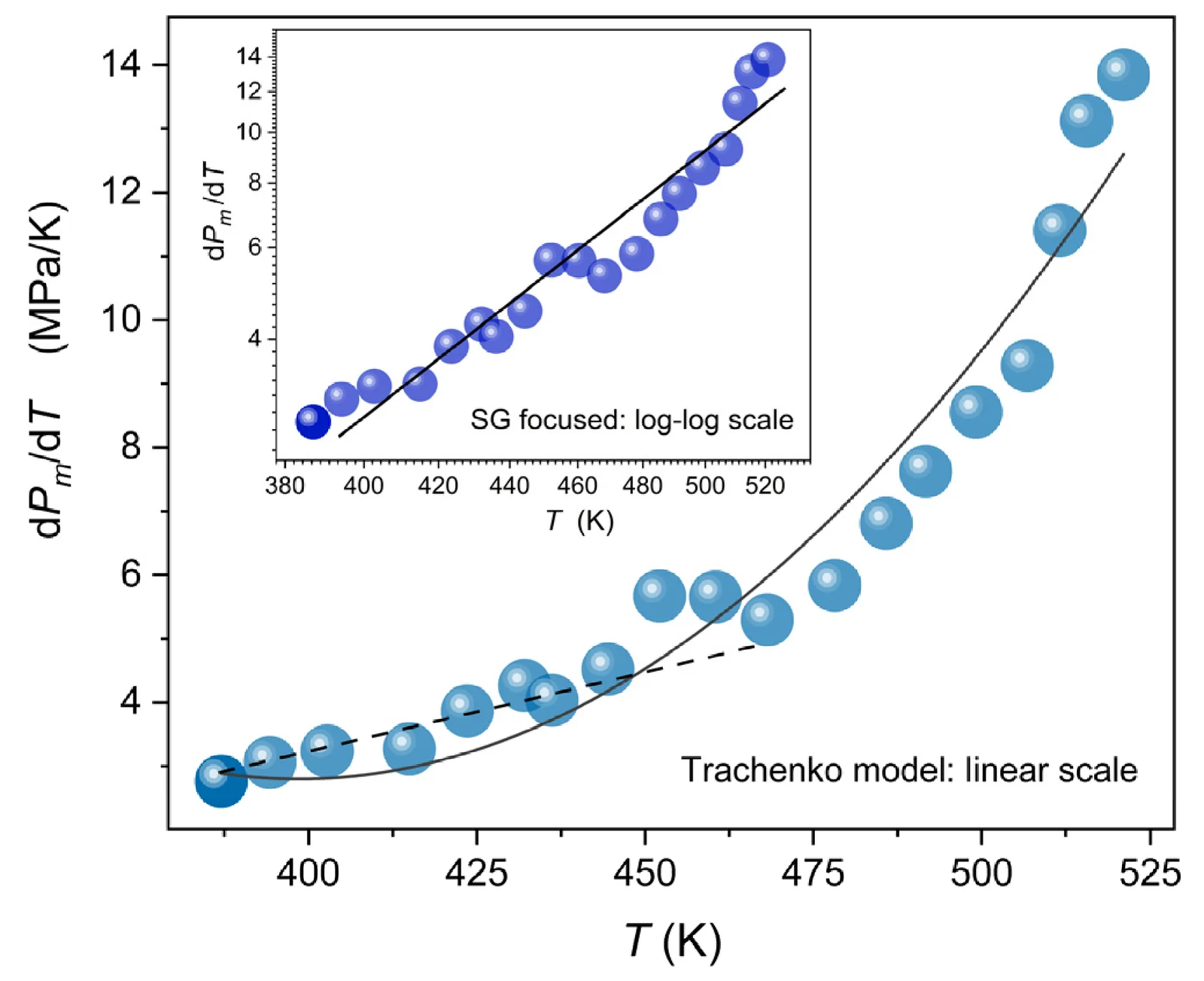
Pressure dependence of the melting temperature in branched polyethylene. The portrayal via the extended SG-type equation (Equation (8)), with $\left(-\pi \right)=-65\;MPa$ and b=0.18. The reference melting under atmospheric pressure $T_{m}=387.8\;K$.

Figure 5 presents evidence of changes in the melting temperature for a branched polyethylene [77,78]. It is applied to form highly flexible, processable, and transparent films commonly used in flexible packaging, agricultural films, and wire insulation. In manufacturing and processing, knowledge of the impact of pressure is essential. Figure 5 presents $T_{m}(P)$ data related to the widest range of pressures ever reported, fairly scaled by the Drozd-Rzoska equation (Equation (8)) with no damping term (π=∞, or c=0), validated by the derivative-based analysis linked to Equation (9). The result of the latter is shown in the inset in Figure 5.

**Figure 6:**
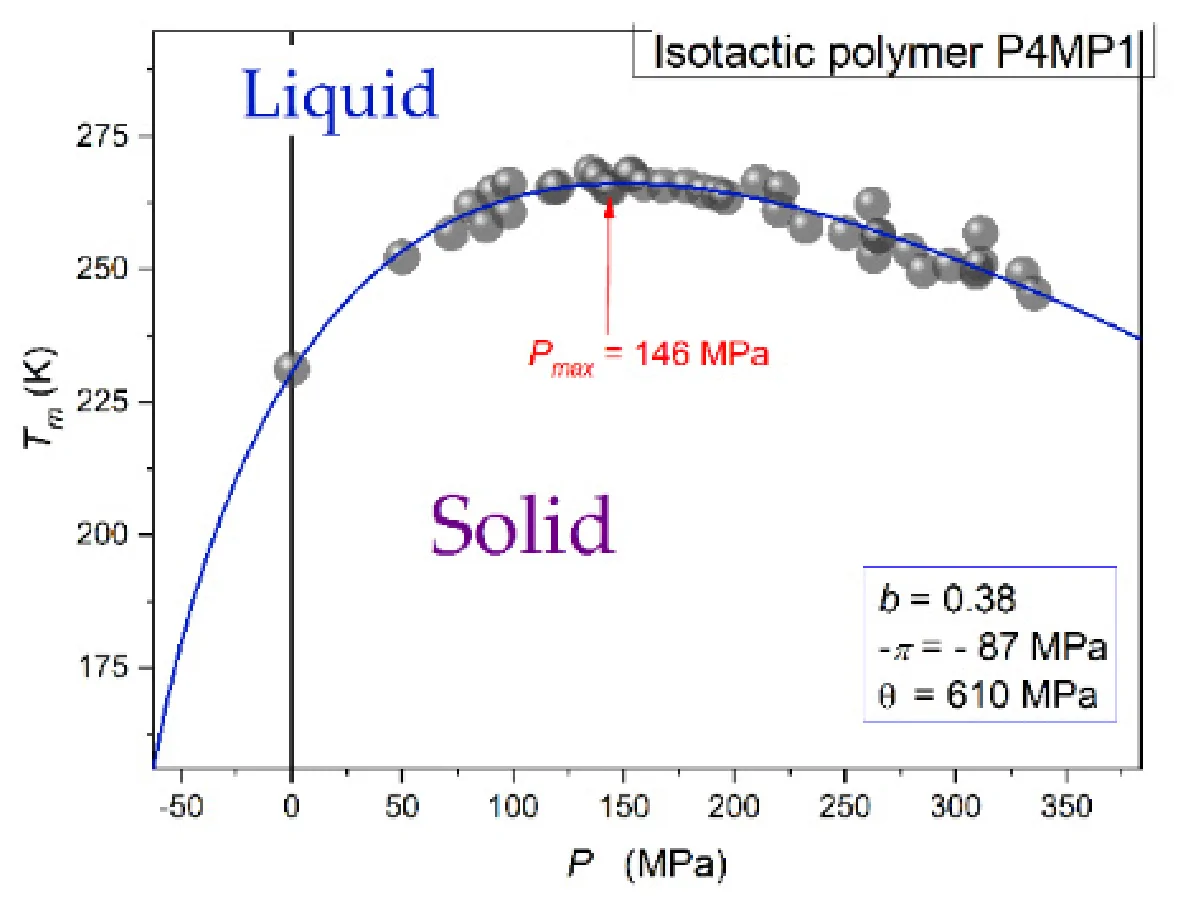
The evolution of melting temperature in poly(4-methyl-pentene-1): isotactic P4MP1 polymer, with parameterization via Equations (8) and (10). Note the extension into the negative-pressure domain, based on data from ref. [79].

Figure 6 shows the unique case of melting-temperature changes under compression in poly(4-methyl-1-pentene) (P4MP1), a semi-crystalline polymer with a crystalline component (~60%) in a tetragonal phase [79]. The parameterization via Equation (8) yields a fair scaling of empirical data, with a maximum at a surprisingly low pressure $P_{max}\approx 146\;MPa$, and the extension into the negative-pressure domain.

When cooling rod-like compounds and liquid-crystalline (LC) materials, mesophases can appear between the isotropic liquid phase and the crystalline solid. For such systems, a specific melting/freezing discontinuous transition associated with a single symmetry element can occur [80,81].

This is not the only unique feature of this phase transition. Also worth stressing is the invariance of the single-molecule primary relaxation time along the melting curve [82,83]. Recalling the Debye–Stokes–Einstein (DSE) relation [84], linking the molecular relaxation time and viscosity, the same invariance along the melting curve should be expected for viscosity. We stress this issue because, for the standard melting line in the *P-T* plane, there is explicit evidence for viscosity changes along the melting curve in ‘standard’ materials where any invariance is absent [22]. It is also notable that for the isotropic liquid–nematic (I-N, the simplest liquid crystalline mesophase) transition, thermal hysteresis does not appear, i.e., the melting temperature $T_{m}$ on heating from the ordered phase and the freezing temperature $T_{f}$ on cooling from the isotropic liquid phase coincide: $T_{m}=T_{f}$ [80,81]. For the melting and freezing in ‘standard’ materials, the hysteresis between phase transition temperatures detected on cooling and heating is the inherent feature, namely, $T_{m}\gg T_{f}$ [85,86]. The hallmark of the LC-mesophase symmetry-limited melting is a weakly discontinuous phase transition nature, i.e., discontinuity metrics like ΔS, or ΔV, are a few times smaller than for standard liquid–solid (crystal) melting. Moreover, long-range pretransitional effects [80,81,87,88,89,90,91,92] assist such transitions. Their manifestation is particularly simple, independently of the nature of the LC-mesophase type, for the low-frequency Nonlinear Dielectric Effects (NDEs) in the isotropic liquid phase [81,87,88,89,90,91]:

(11) $$\frac{{\Delta \varepsilon }^{E}}{E^{2}}=\frac{A}{T-T^{*}}\;\Rightarrow \;{\left(\frac{{\Delta \varepsilon }^{E}}{E^{2}}\right)}^{-1}=AT-AT^{*}=AT-b\mathrm{for}\;T>T_{I-LC}-{\Delta T}^{*}$$

where ${\Delta \varepsilon }^{E}=\varepsilon \left(E\right)-\varepsilon$ describes changes in dielectric constant due the impact of the strong electric field; $T^{*}$ is the extrapolated temperature of a hypothetical continuous phase transition, $T_{I-LC}$ is the isotropic liquid–LC discontinuous (melting) transition, and ${\Delta T}^{*}$ is the metric of the discontinuity.

**Figure 7:**
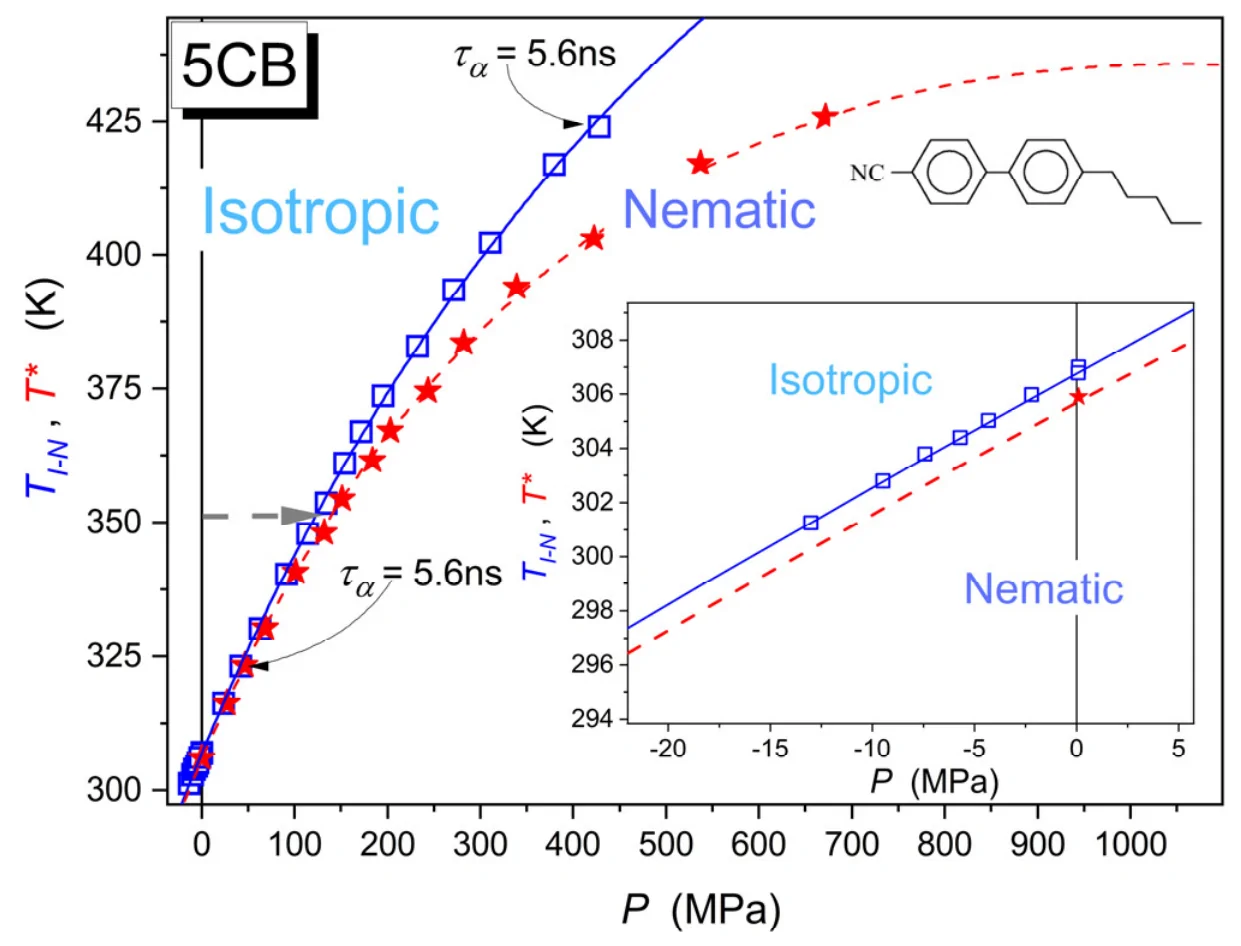
Pressure changes in the isotropic liquid–nematic discontinuous transition temperature (square) and the coupled extrapolated continuous/pseudospinodal temperature (stars). The portrayal is related to Equation (8). The inset focuses on the behavior in the negative-pressure domain. $T_{I-N}(P)$ scaling is related to π=−320 MPa, b=0.51, and θ=4.0 GPa, and π=−265 MPa, b=0.44, and θ=3.0 GPa for $T^{*}\left(P\right)$.

The evidence for NDE pretransitional behavior, including its pressure-related counterpart, is presented in references [87,88,89,91], for I-N, I-SmA, and I-SmE transitions. Figure 7 presents the pressure evolution of the isotropic–nematic phase transition temperature in 4-cyano-4′-pentylbiphenyl (5CB, pentylcyanobiphenyl), one of the most classic LC materials [80]. The nematic phase is the simplest LC mesophase, associated with only one element of symmetry freezing: uniaxial ordering. The plot also shows pressure changes in $T^{*}$, related to a hypothetical continuous transition, indicated in Equation (11). Comparing the discontinuities of the I-N and N-solid transitions in 5CB, one can estimate the following values, recalculating those given in ref. [92]: ${\Delta S}_{I-N}=35\;JK{kg}^{-1}$ and ${\Delta S}_{N-S}=380\;JK{kg}^{-1}$ at P=0.1 MPa. The I-N discontinuity entropy change for the I-N transition is almost 10× lesser than for the nematic–solid (N-S) transition.

Both $T_{I-N}\left(P\right)$ and $T^{*}\left(P\right)$ changes are fairly scaled by Equation (8), indicating possible maxima at $P_{m}^{max}\approx 1.75\;GPa$ and $P_{max}^{*}\approx 1.05\;GPa$. For $T_{I-N}\left(P\right)$ changes, the experimental evidence was extended into the negative-pressure domain, using the Berthelot method [67]. It is still well captured by Equation (8), experimentally validating the scaling extended in the negative-pressure domain.

In Figure 7, molecular time scales, determined from the primary (*alpha*) relaxation time detected in Broadband Dielectric Spectroscopy (BDS) studies (see Methods section), are also indicated for the I-N transition on compression up to P≈425 MPa.

This time scale remains constant along the single-element-of-symmetry (uniaxial orientation) melting curve (I-N transition). In earlier studies, such evidence was shown only up to P~150 MPa [82,83]. One can recall here the vividly discussed question of a lower bound value for viscosity—as well as primary relaxation time—in liquids (of course ‘standard’, not supercooled, liquids). This is the famous ‘Purcell question’, remaining a significant research challenge [93,94,95,96]. Viscosity and the primary relaxation time are coupled by the Debye–Stokes–Einstein relation [84]. So, the invariance, for a given material, of primary relaxation time along the melting curve in the *P-T* plane also means invariant viscosity. Results of this report and refs. [22,82,83] suggest different patterns for Soft Matter, symmetry-limited melting curves (like 5CB), and ‘standard’ ‘Hard’ materials, for which melting is associated with a large number of symmetry elements. This introduces a new issue in the ’Purcell question’ discussion: the role of molecular symmetry and the impact of compression along the melting line.

## 3. Discussion

The above discussion showed the concept of the coherent description of systems showing three basic patterns for the impact of pressure on the melting temperature, related to $dT_{m}/dP>0$, $dT_{m}/dP<0$ and the $T_{m}(P)$ maximum. Due to the scaling by the Drozd-Rzoska equation (Equation (8)), coupled with the ‘*bottom-up*’ protocol defined by Equations (9) and (10), a coherent description linking these patterns is possible [23,68,69]. The introduction of negative pressures into the analysis is particularly worth stressing.

In this section, we discuss some supplementary but also significant issues related to the pressure dependence of melting temperature.

Recently, a new universalistic scaling relation for the melting curve, which should hypothetically apply to any type of material, has been proposed. Trachenko [43] developed the new theory, finally showing that the melting line in the *P-T* plane can be expressed by a simple parabolic function (Equation (7)). The new theoretical insight significantly focused on the liquid side of $T_{m}$, instead of the solid (crystal) side dominated in earlier studies [22,24,25], as noted in the Introduction. Trachenko stressed [43]: ‘*it is not only universal but also is governed by fundamental physical constants such as the Planck constant and electron mass and charge …melting, despite its complexities, exhibits a fundamental unity across diverse systems, from noble gases to metals*’. The basic output conclusion of the Trachenko model was the new parabolic relation [43]:

(12) $$P_{m}=P_{0}+\frac{a}{2b}T+\frac{\Delta }{2b}T^{2}=D_{1}+D_{2}T+D_{3}T^{2}$$

where $D_{i}$ is the symbolic presentation of constant parameters;$\;a=2\left(\alpha_{tL}-\alpha_{t}\right){\left(r-1\right)}^{-1}$, $b=\left(r/B_{L}-1/B_{S}\right){\left(r-1\right)}^{-1}$, $d=\left(\alpha_{tL}^{2}B_{L}r-\alpha_{tS}^{2}B_{S}\right){\left(r-1\right)}^{-1}$ , $r=V_{L}/V_{S}$, and $\Delta =2bd-a^{2}/4$; in these relations, B is the bulk modulus. $\alpha_{t}$ denotes the thermal expansion coefficient; V is the volume; and indices ‘*_L_*’ and ‘*_S_*’ denote the liquid and solid sides of the melting temperature.

The above scaling equation was derived by implementing the Phonon Theory of Liquid Thermodynamics for the liquid state near the melting transition, the basics of which Trachenko presented in refs. (2023, [43,97,98,99]). This model concept essentially focuses not on entropy but on latent heat (enthalpy) change.

Although ref. [43] was published only recently, it has already become a significant inspiration for further theoretical explorations [50,51,52,53,54,55,56,57,58,59,60]. Recently, Papathanassiou [60] considered an alternative model focused on variable-pressure fusion enthalpy. Following recent experimental findings on heat capacity in the solid near melting, an analytically solvable model for the melting line in *P-T* space was derived by accounting for the anharmonic, volume-dependent nature of the isobaric heat capacity and the enthalpy of fusion. The resulting model scaling equation could be well approximated by a parabolic function. The author of ref. [60] concluded: ‘*This striking agreement* (with the Trachenko model)*, arising from completely distinct theoretical starting points, establishes the universal parabolic nature of melting curves and offers a unified perspective on the thermodynamics of the solid-liquid transition*.’ Papathanassiou [60] stressed the significance of the experimental validation presented by Trachenko [43] for validating the new melting model.

Notably, the discovery reported in ref. [43] immediately had a huge impact on PR-type science-focused info channels [44,45,46,47,48,49]. It was announced as ’*Breakthrough in melting point prediction: over 100-year-old physics problem solved…*’ [44,45] or ‘*physicists do not always know how to predict at what temperature and pressure a material will melt. He* (Trachenko) *has now derived an equation that can be used to make such predictions for a vast range of substances*.’ [46]. Nevertheless, Orf, while emphasizing the importance of ref. [49], also stressed the need to validate this result: ‘*A new study put forward a theory that leverages Planck’s constant and electric properties instead of entropy to developed a long-sought-after equation…..While a promising solution to the long standing problem, a theory needs to put up a rigorous testing*’.

In ref. [43], the experimental validation, which showed a superior fit to the parabolic equation (Equation (12)), was identified as a particularly significant argument supporting the model’s reasoning and the discovery of the universal pattern scaling. This is presented in Figure 1 of ref. [43], where the upper part ideographically shows $T_{m}$ changes for He, H, Ar, H_2_O, and Fe over a wide range of pressures, with relatively strongly pronounced nonlinearity, ranging from atmospheric pressure to even over 20 GPa for helium and 60 GPa for iron.

However, in ref. [43], experimental validation is shown only for a small part of these data, limited to approximately 3 GPa. The exceptions are H_2_O, where the tested range is from ~2.2 GPa to 4.2 GPa, and iron, where this domain reaches 30 GPa, i.e., half the range of the reference data. For each ‘validation case’ tested in ref. [43], the nonlinearity of $T_{m}(P)$ changes is ‘weak’. For such range-limited data, one can (always) expect a fair portrayal by a second-order polynomial (‘parabolic’, Equation (12)) as well as by the basic SG equation (Equation (4)). Both dependencies for $P_{m}(T)$ scaling have the same number of adjustable parameters.

Hence, a question arises for the ultimate reliability of the experimental validation of the ‘parabolic’ model scaling presented in ref. [43]. Reliable experimental validation is the *sine qua non* condition for The Scientific Method [100], especially since in subsequent works referring to Trachenko’s ‘universal’ scaling, the experimental validation presented in ref. [43] is an important argument [60].

For the $P_{m}(T)$ changes considered in ref. [43], fitting via a second-degree polynomial (‘parabolic’) always yields excellent scaling quality. However, Bridgman, the ‘father’ of High-Pressure Physics, also used a second-degree polynomial in this type of analysis, but only as a tool for formal and limited pressure-range description [101,102].

To address the challenge of validating the Trachenko ‘parabolic’ model [43] and ultimately comparing it with the standard Simon–Glatzel scaling, one of us (A. Drozd-Rzoska) proposes the following derivative-based analysis:

(13) $$P_{m}{=D}_{1}+D_{2}T+D_{3}T^{2\;}\;\Rightarrow \;\frac{dP_{m}\left(T\right)}{dT}=D_{2}+D_{3}T$$

where the right-hand part shows the proposed derivative-based transformation of empirical data.

For the basic Simon–Glatzel dependence (Equations (4) and (5)), one can consider

(14) $$P_{m}=-A^{\prime }+B\times T^{b}\;\Rightarrow \;\frac{dP_{m}\left(T\right)}{dT}=bB\times T^{b-1}\;\Rightarrow \;\Rightarrow \;log\left(\frac{dP_{m}\left(T\right)}{dT}\right)=log\left(bB\right)+\left(b-1\right)logT$$

Notably, they are essentially different functional forms of Equations (13) and (14).

**Figure 8:**
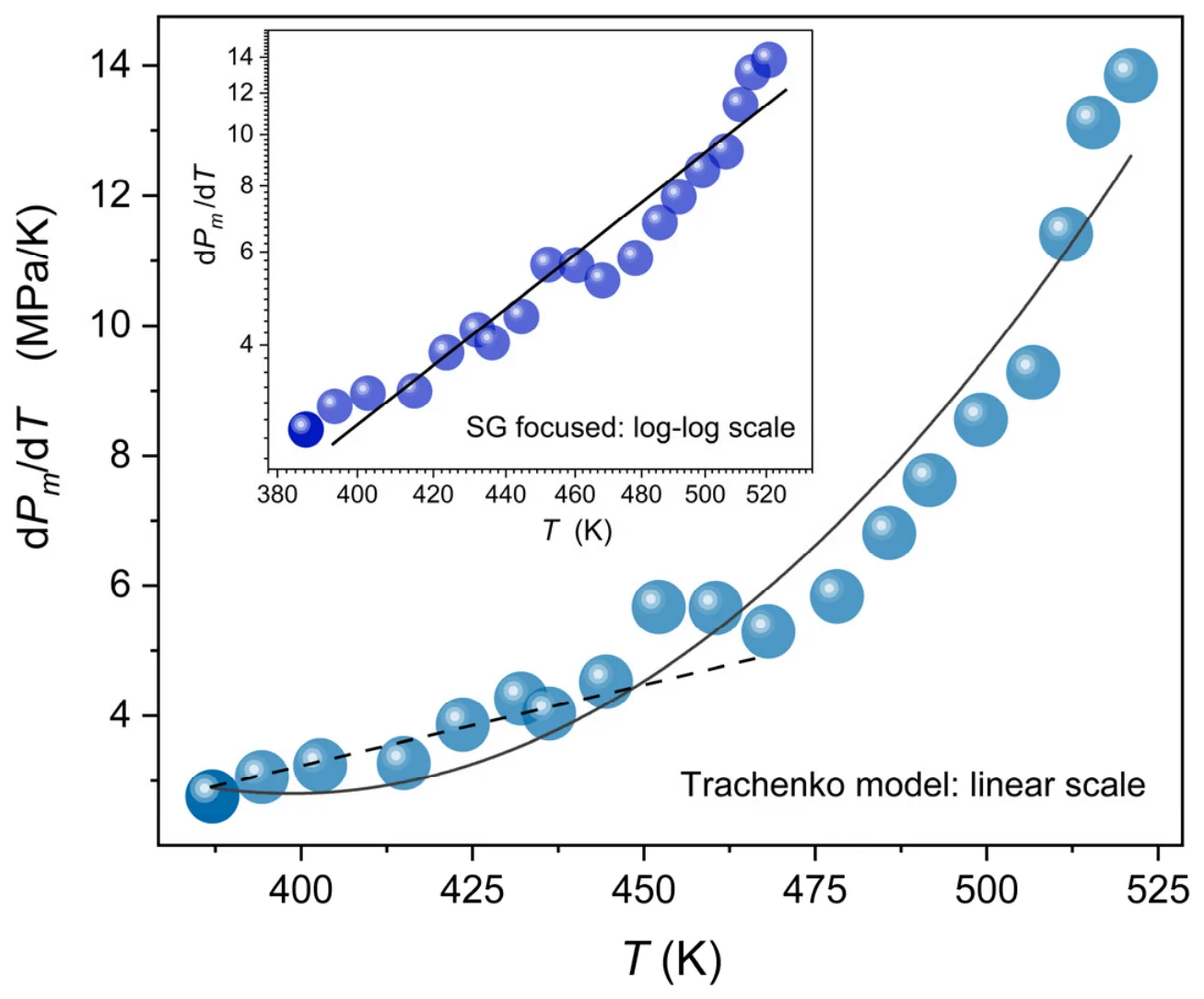
Results of the derivative-based analysis focused on validating the SG-type scaling equations (Equations (13) and (14)) shown in the inset and in the central part of the new, hypothetically universalistic, Trachenko model [43]. The domain of a validated implementation is related to the linear behavior. The plot is based on $T_{m}(P)$ data for polyethylene, shown in Figure 5.

Following Equation (13), transformed experimental data in the scale $dP_{m}/dT$ vs. T should follow a linear behavior, to validate scaling via the Trachenko model [43]. To validate Simon–Glatzel-type scaling, the same data should be presented on a log–log scale, as defined by Equation (14). Such behavior is tested in Figure 8. Its central part relates to the Trachenko scaling [43] distortion-sensitive validation test defined by Equation (13). The pattern is essentially nonlinear. The approximate linear behavior can be considered in the range ~200 MPa, similar to the one tested in ref. [43]. The explicit linear behavior in the full range of pressures, up to ~700 MPa, takes place for the SG-type scaling, tested via the derivative-based scaling defined by Equation (14). It is presented in the inset in Figure 8.

These results provide an explicit negative verification of the universalistic ‘parabolic’ scaling of $P_{m}\left(T\right)$ changes as described by Trachenko ‘parabolic’ scaling [43], and defined by Equation (12). Notably, the functional patterns of $dP_{m}/dT$ changes defined in Equations (13) and (14) exclude the simultaneous validity of Simon–Glatzel and Trachenko scaling equations, heuristically suggested in ref. [43].

Concluding, the above results indicate that the models developed in refs. [43] and [60], which suggest the ‘universal, parabolic’ scaling, are not validated with respect to empirical data distortions sensitive analysis.

It is worth noting that the ‘parabolic scaling’ also does not address systems associated with $T_{m}(P)$ maximum or $dT_{m}/dP<0$.

This report largely addresses melting in Soft Matter systems, which are associated with inherently weak intermolecular or inter-assembly interactions—qualitatively weaker than in standard solid materials (‘Hard Matter’). This difference is primarily reflected in the parameters in the scaling relations describing $T_{m}(P)$ behavior (Equation (8)). Notable is the significantly smaller value of the parameter Π in Soft Matter, which also means a qualitatively smaller value of $\left(-\pi \right)$, the stability limit.

**Figure 9:**
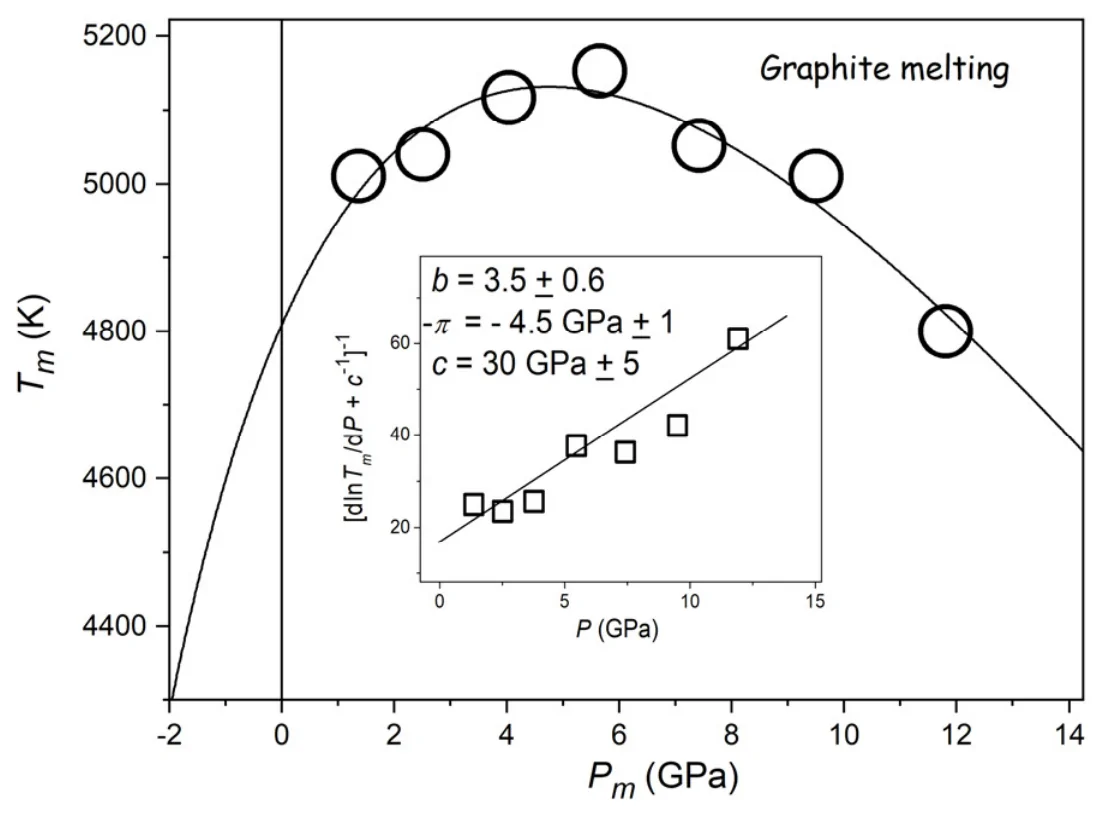
Melting curve in *P-T* plane for graphite, with the maximum at P=4.8 GPa. The inset shows the derivative-based transformation of data (Equation (8)), validating the portrayal by Equation (10) and yielding optimal values of parameters. The plot recalls empirical data from ref. [103]. Diamond is a symbolic material of immense hardness. Graphite’s specific softness, in turn, is experienced in everyday practice, for example, when drawing with a pencil. This results from the specific structure of graphite, organized into strong bonds within layers and very weak bonds between layers.

**Figure 10:**
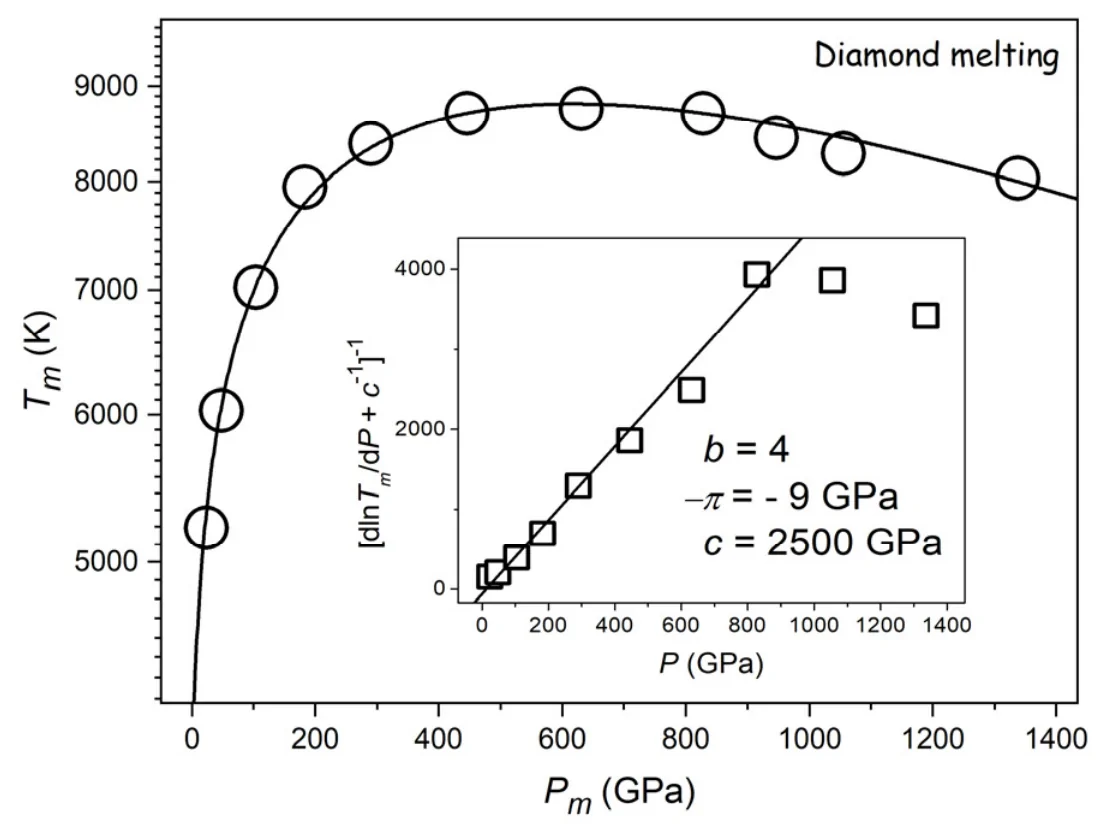
Melting curve in *P-T* plane for diamond, with the maximum at P=630 GPa. The inset shows the derivative-based transformation of data (Equation (8)), validating the portrayal by Equation (10) and yielding optimal values of parameters. The plot recalls empirical data from refs. [103,104].

This phenomenon can be illustrated by comparing the melting curves of graphite and diamond, two allotropic forms of carbon. They are presented in Figure 9 and Figure 10, scaled via the Drozd-Rzoska equation (Equation (8)). The insets show the preliminary analysis via Equation (10), with parameters obtained on this basis. The pattern for diamond is explicitly ‘Hard Matter’-type. But for graphite, it shows features of the Soft Matter type. This can be related to the particular crystal structure of graphite, namely strong interatomic bonds in planes and very weak bonds between these planes. This can be recognized as a specific combination of ‘Hard Matter’ and ‘Soft Matter’ features. Finally, the parameters in $T_{m}\left(P\right)$ scaling relation are close to those observed in Soft Matter. It also demonstrates the crucial importance of intermolecular/interatomic interaction in characterizing the changes in the melting curve under pressure.

The first words of de Gennes’ Nobel Prize lecture [62], ‘*What does Soft Matter mean?*’, can be recalled here as a memento.

## 4. Materials and Methods

Perkin first observed the impact of pressure on the melting point as early as 1826 [105]. However, for contemporary research, Bridgman’s work is particularly important [101,102,106,107]. As early as 1911, he demonstrated the possibility of detecting the melting point under pressure through in situ measurements of electrical resistance [106,107]. In 1920, Bridgman implemented a second detection method by lowering a cylindrical crucible at a constant velocity along the axis of a vertical tubular furnace held at a constant temperature [101,102]. This method also allows for determining volume changes during transition, and thanks to the C-C relationship, also ΔH or ΔS, with at least preliminary knowledge of $T_{m}(P)$. The authors of this work, based on their long experience in pressure research, can point out certain problems of the second method related to increasing mechanical resistance during compression, which limits accuracy. In situ measurement (the first method) provides much more accurate estimates. Bridgman was also a pioneer in developing laboratory high-pressure techniques, introducing solutions still used today [106,107]. For his remarkable achievements, Bridgman received the Nobel Prize in 1946 “*for the invention of an apparatus for producing extremely high pressures, and for the discoveries he made therein in the field of high-pressure physics*.” [108].

**Figure 11:**
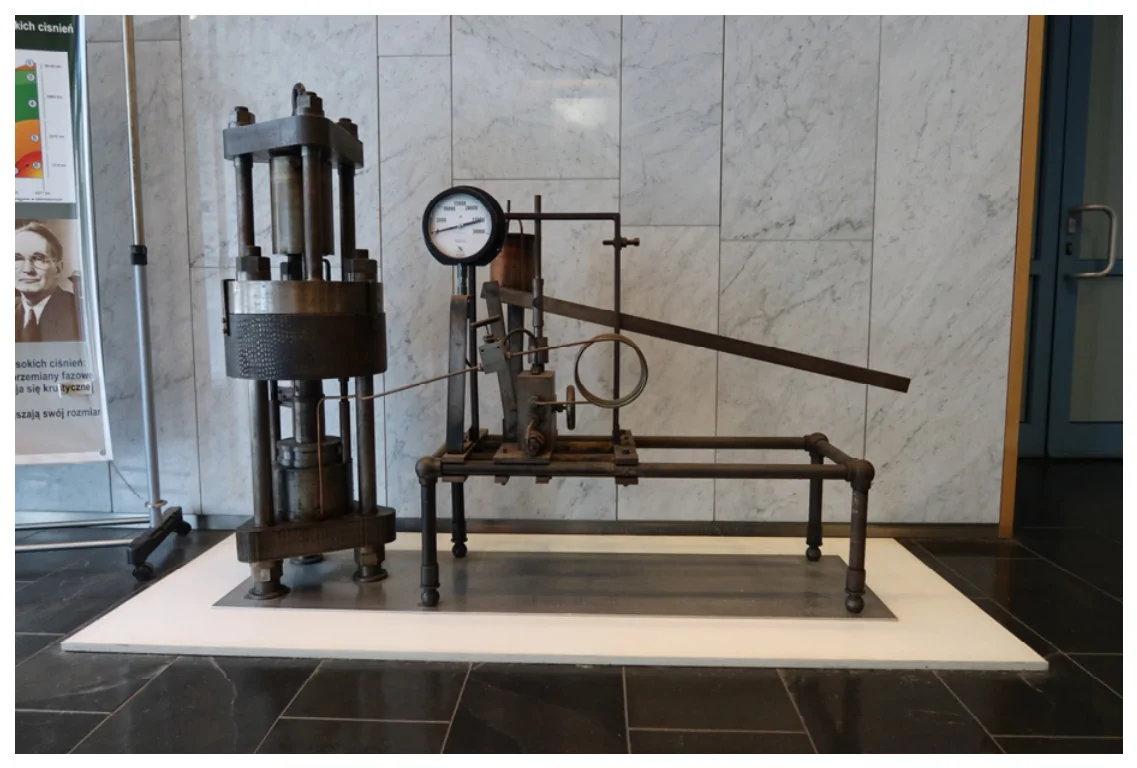
Original high-pressure apparatus/processor by Percy W. Bridgman, Nobel Prize in Physics 1946. It has been on display for a few decades in the Hall of the Institute of High-Pressure Physics of the Polish Academy of Sciences, Warsaw, Poland.

Figure 11 shows a unique Scientific Relic: Bridgman’s original high-pressure apparatus, still ready for in situ high-pressure measurements. It is on display in the Hall of the Institute of High-Pressure Physics of the Polish Academy of Sciences (IHPP PAS) ‘Unipress’ in Warsaw, Poland. It appears there due to the initiative of Prof. Sylwester Porowski (co-author of this report), who in the late 1960s held a research fellowship at Harvard University, including the former P.W. Bridgman laboratory.

**Figure 12:**
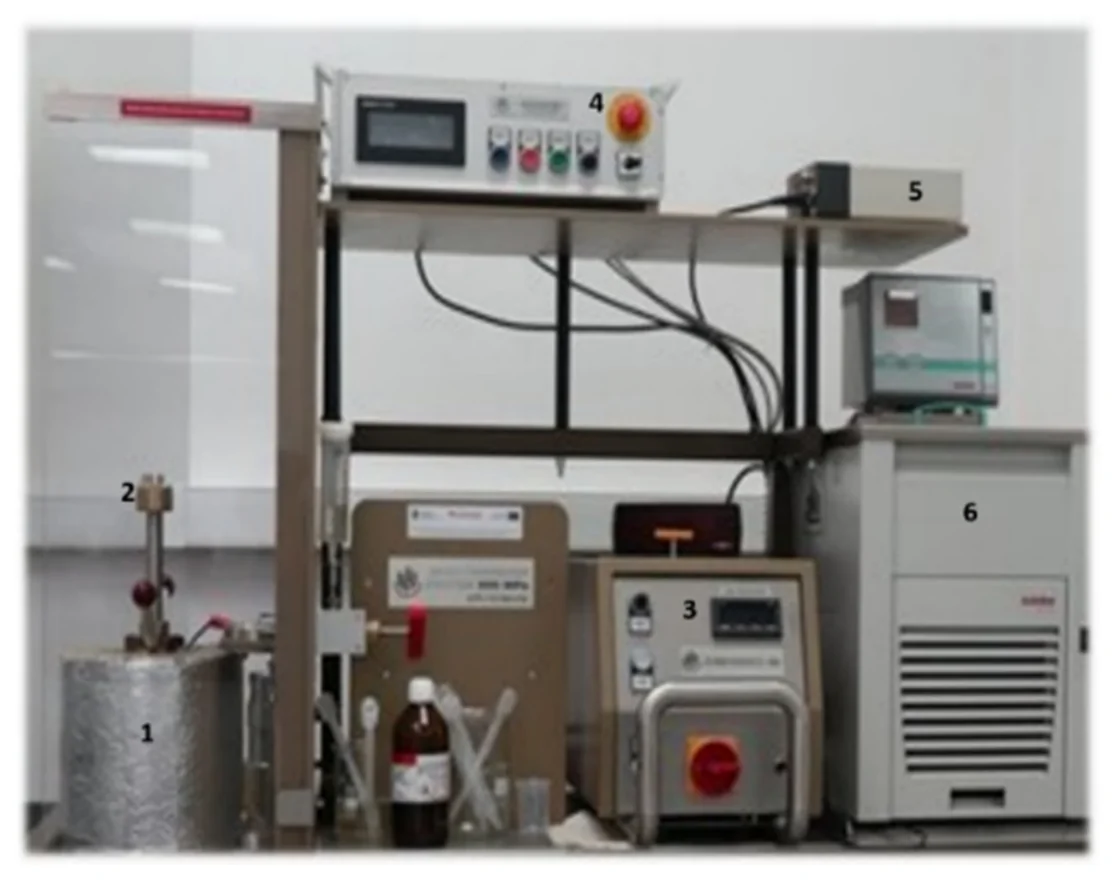
High-pressure (up to 0.9 GPa, −30 °C < *T* < 15 °C, V=20 mL) setup for in situ Broadband Dielectric Spectroscopy (BDS), DTA, and density tests. (1) High-pressure chamber (HPC), (2) output for links with external monitoring devices, (3) HP pump, (4) the pump controller, (5) pressure meter, and (6) large-volume thermostat with external circulation for the jacket surrounding HPC. The facility is available in IHPP PAS ‘Unipress’, X-Press-Matter Lab, Warsaw, Poland: (https://young4softmatter.pl/).

This report explores a few sets of reference $T_{m}\left(P\right)$ data recalling authors’ ‘melting data’ first reported in refs. [23,91] and new ones obtained by detecting step changes in dielectric properties, such as the dielectric constant. The uniqueness of the applied high-pressure setup lies in the ability to make controlled, precise changes in pressure during both compression and decompression in the isothermal case, and during cooling and heating. This is matched with computer-controlled temperature changes and advanced data registration from in situ measurements. Figure 12 shows the experimental facility used, available at the X-PressMatter Lab of IHPP PAS in the Innovation Park in Celestynów, near Warsaw. For monitoring, the authors used a Novocontrol Alpha BDS (Broadband Dielectric Spectroscopy) impedance analyzer. Samples were placed in a specially designed flat-parallel capacitor made from gold-coated Invar, within a module that guarantees complete isolation between the tested sample and the pressure-transmitting medium in the high-pressure system and enables tests under both compression and decompression.

The crucial challenge in pressure-related experiments is completely isolating the tested sample from the pressure-transmitting medium. It is particularly significant for Soft Matter materials, where even a tiny amount of contamination can substantially change properties, including phase transition temperatures. The next challenge is the need to operate both in compression—the standard experimental direction—and in decompression, still hardly available in labs. The latter is essential for research related to melting-pressure detection. These challenges limit high-pressure research, especially in the absence of reliable commercial solutions.

**Figure 13:**
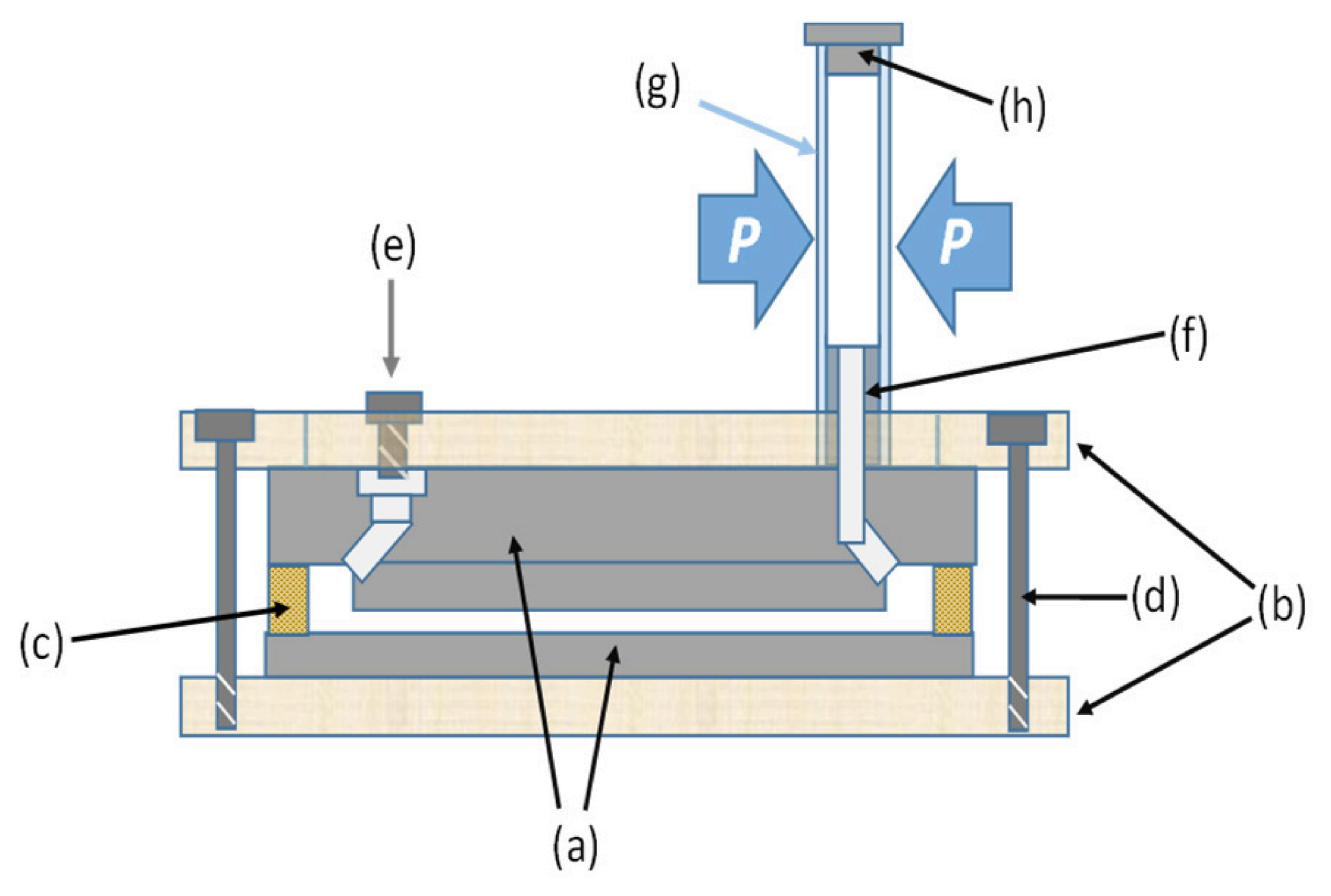
A module dielectric capacitor for tests inside a high-pressure chamber, made from Invar to avoid the impact of temperature changes: (*a*) capacitor plates (diameter, 15 mm; gap, 0.1–0.5 mm) connected to the passages in the pressure chamber enabling further connection to the impedance analyzer (BDS), (*b*) clamps fastening the plates, (*c*) circular spacer made of high-quality quartz, (*d*) mounting screws, (*e*) screw closing the hole enabling the introduction of a sample using a syringe, (*f*) fastening element for flexible tubes, (*g*) flexible tube transmitting pressure during compression and decompression, (*h*) closing the tube transmitting pressure.

Figure 13 presents the new capacitor concept for dielectric studies under pressure developed for the studies reported above. A unique feature is the pressure-transmitting element based on a cheap, readily available, and easily replaceable flexible tube. It ensures effective pressure transmission to the sample during compression and decompression and enables compression/decompression tests for subsequent isotherms without risk of any contamination. In a single measurement cycle, for the same sample, it is possible to detect the complete $T_{m}(P)$ curve across the full required pressures. This makes studies time- and cost-efficient, solving one of the experimental challenges in high-pressure research.

**Figure 14:**
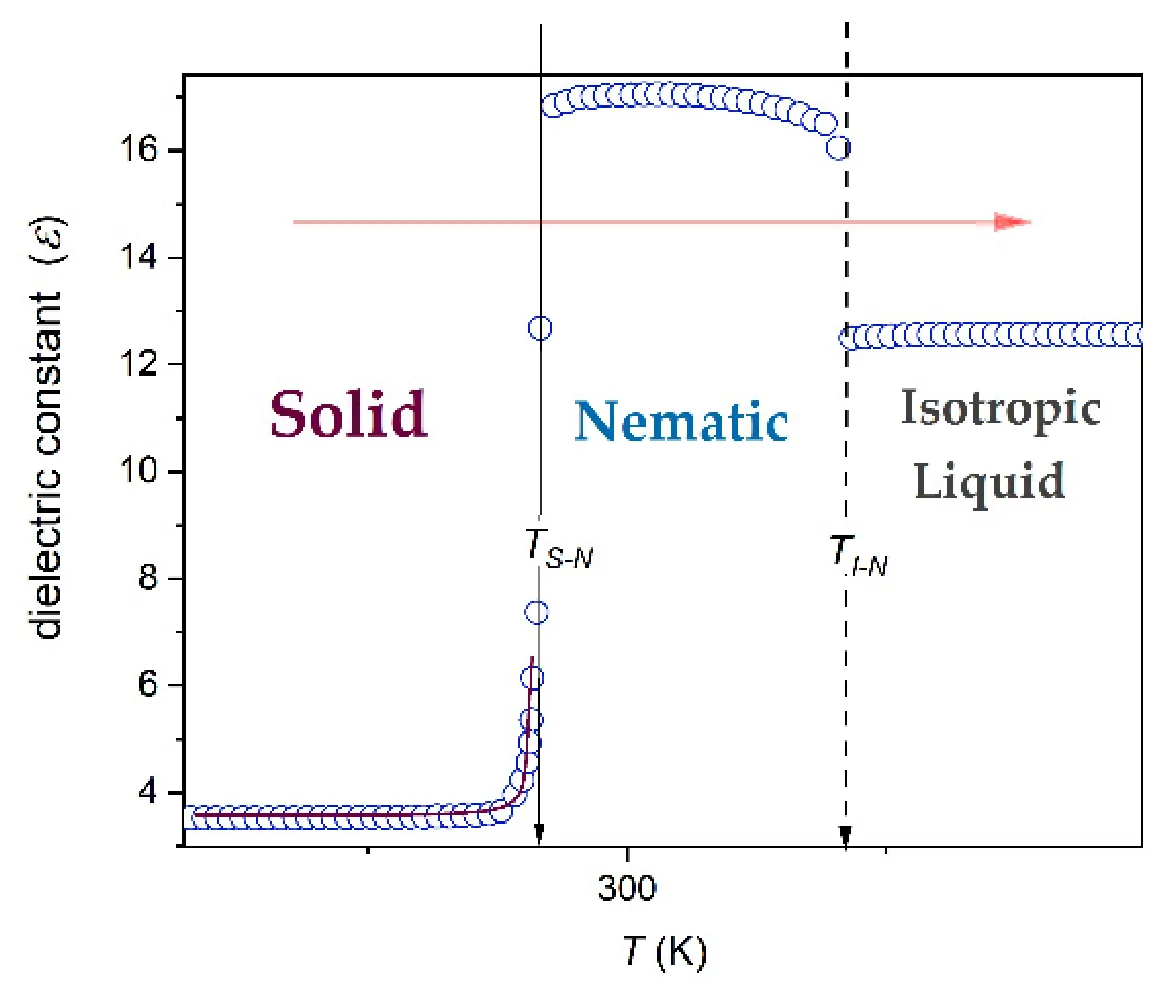
The sequence of phase transitions in liquid crystalline 4-cyano-4′-pentylbiphenyl (5CB), a material for which the evidence of symmetry-selected melting temperatures ($T_{S-N},T_{I-N}$) and pressure dependences is presented. The horizontal light-red arrow shows the direction of temperature changes. Vertical arrows indicate subsequent discontinuous phase transitions. The final section of the report discusses the premelting effect.

Figure 14 demonstrates changes in dielectric constant during phase transitions in liquid crystalline, rod-like pentyl cyanobiphenyl (5CB). Upon heating from the solid phase, the first step is melting to the fluid nematic phase ($T_{S-N}\;$). Subsequently, melting associated with only one element of symmetry (uniaxial, orientational ordering) takes place from the nematic to the isotropic liquid phase ($T_{I-N}$). 5CB was obtained due to the courtesy of the LC team at the Warsaw Military University (Poland). Polymeric compounds were purchased from Merck. They were dried and degassed thoroughly before testing.

The significance and experimental convenience of the dielectric methods used for high-pressure studies are worth stressing. Figure 14 shows the effectiveness of this detection, with a well-defined and ‘strong’ change in dielectric constant across the melting transition. Notably, the premelting effect appears in the solid phase. For these studies, pressure was transmitted via Plexol, an intermediary liquid, generally advised as the optimal medium in high-pressure studies for this purpose. It should also be stressed that pressure was applied to the sample isotropically, following the pattern commonly named ‘hydrostatic’ pressure.

The premelting effect is considered a significant prerequisite for melting but is, in fact, impossible to parameterize in a reasonable way [109,110,111,112,113,114,115,116]. Changes in the dielectric constant are an unusual exception, where the fair scaling via the following relation is possible [115,116]:

(15) $$\varepsilon \left(T\right)=C+\frac{A}{T-T^{+}}$$

where the singular temperature $T^{+}=T_{m}+0.8K$.

The similarity of this relation to Curie–Weiss or Mossotti Catastrophe scaling is not accidental, as initially explained in refs. [115,116]. Melting transition studies using BDS offer the opportunity not only to accurately detect melting transitions but also to identify an unusual phenomenon that warrants further investigation. Notably, the premelting effect also appears in pressure studies on decompression from the solid phase [115].

Melting temperature is a material constant characterizing the material, determined on heating from the solid phase. For the presented studies, it was determined for three ‘control scans’. It was possible because the new capacitor concept presented in Figure 13 enabled repeatable tests for compression and decompression, as well as for subsequent isotherms, without any risk of contamination—a significant problem in high-pressure studies of Soft Matter systems, where even a tiny impurity can strongly influence properties. The capacitor in Figure 13 requires only ~0.5 ccm of sample, but ‘bulk’ conditions for the sample were preserved.

## 5. Conclusions

This report focuses on the melting curve/line in the pressure–temperature plane, with an emphasis on the Simon–Glatzel (SG) and Rein–Demus–Kechin (RDK) scaling relations. It is shown that model extension by the Drozd-Rzoska equation [23,68], explicitly including the negative pressure, allows for a coherent description of $T_{m}\left(P\right)$ evolution in systems where $dT_{m}/dP>0$, a $T_{m}(P)$ maximum occurs, and $dT_{m}/dP<0$. The latter is related to the $T_{m}(P)$ maximum hidden in the negative-pressure domain.

An important issue is the linearized derivative-based analysis defined by Equations (9) and (10), which allows for a preliminary determination of the pressure/temperature range in which a given scaling description can be applied, and for determining the optimal parameter values in scaling relations. They can be substituted directly into the scaling relation, thereby avoiding nonlinear fitting. Nonlinear fitting for an ad hoc-selected range of pressures can portray experimental data, but obtained parameters can only be ‘effective’

The reported analysis is validated against $T_{m}(P)$ empirical data in a few significant systems, yet underdiscussed. The first group comprises basic materials essential to the semiconductor industry: gallium nitride, germanium, and silicon. In the latter two cases, the maximum of the $T_{m}(P)$ curve in the negative-pressure region was identified, and its parameters were determined. Next, the particularity of systems in the Soft Matter category was highlighted. The relatively weak inter-element interactions that characterize it lead to high sensitivity to exogenous impacts, including pressure. In the P4MP1 polymer, the $T_{m}(P)$ parameters were demonstrated and determined, in the unusual situation where the maximum of this curve, i.e., the $dT_{m}/dP>0\;⟶\;dT_{m}/dP<0$ crossover, appears for a surprisingly low pressure of T~160 MPa.

For n-pentylcyanobiphenyl (5CB), a classical liquid crystalline material with the nematic mesophase, it was shown that symmetry-limited melting associated with only one symmetry element, $T_{m}=T_{I-N}(P)$, i.e., uniaxial orientational ordering, is also subject to the scaling described above. This also applies to temperature changes in the hypothetical continuous phase transition $T^{*}(P)$. It is worth emphasizing that validation of such a description in the negative-pressure region for experimentally determined values of $T_{I-N}(P<0)$ was demonstrated [91].

The final section of the report addresses the recent discovery of a hypothetically universal parabolic scaling of the melting curve $P_{m}(T)$ by Trachenko [43]. This result is particularly interesting because it is motivated by a new fundamental basis that offers ‘new hope’ for a universalistic cognitive breakthrough in the melting transition cognition. Empirical validation of this result was based on excellent reproduction of empirical data for five materials, but in a limited pressure range [43]. The quality of this fit was comparable to that of the standard Simon–Glatzel relation (Equation (4)). This comparable quality of fit is not surprising, given the relative functional similarity between the SG relation and that introduced by Trachenko [43]. However, the validation test based on the $dP_{m}/dT$ derivative for both relations proposed in the given report is related to qualitatively different validation functions (Equations (13) and (14)) for Simon–Glatzel and Trachenko scaling relations.

The resulting experimental validation test is explicitly negative for the parabolic Trachenko [43] scaling, as evidenced in Figure 8.

A direction for future research can be to conduct such validation tests on other materials. However, there is excellent and extensive experimental evidence validating SG scaling also with preliminary distortion-sensitive and derivative-based analysis focused on dlnT(P)/dP behavior (Equations (9) and (10))—as well as at pressure ranges substantially broader than those discussed in ref. [43].

In summary, SG-type and RDK scaling remain the dominant tools for scaling the melting curve in the *P-T* plane, particularly given the extension proposed by Drozd-Rzoska [23], which explicitly includes the negative-pressure domain in the analysis. We would like to emphasize, however, that the results and discussion presented here do not address the numerous cases in which new phases appear during compression.

The basic results can be summarized as follows:

(*i*) The coherent discussion on $T_{m}(P)$ behavior both for ‘Hard Matter’ (‘standard’ solid state) and Soft Matter systems reveals the importance of the strength of intermolecular interactions; (*ii*) the model picture linking systems with $dT_{m}/dP>0$, $dT_{m}/dP<0$ and the $T_{m}\left(P\right)$ curve maximum, which also shows the relevance of the negative-pressure domain; (*iii*) the ultimate validation test of the recent ‘universal’ scaling of the $T_{m}(P)$ pattern derived by Trachenko [43] against empirical data with new relations enabling ultimate validation; (*iv*) the explicit evidence of the premelting effect with ‘Mossotti Catastrophe’-type scaling (Figure 14 and Equation (15)) is worth stressing.

The results of this work can also be significant for scaling the pressure dependence of the glass temperature $T_{g}$, which can be treated as a specific ‘diffused, melting-type’ transition [117]. In 1998, Andersson and Andersson [118] applied the SG parallel to effectively describe changes in $T_{g}(P)$. Earlier, Turnbull and followers, based on an extensive analysis of experimental data, demonstrated the importance of the $T_{g}/T_{m}$ ratio [119,120,121,122] for the ease of supercooling a liquid to the glass state. Drozd-Rzoska et al. [23,68,69] showed the relevance of the parallel of Equations (8)–(10) for scaling $T_{g}(P)$ evolution, as well as, for the first time, systems with the $T_{g}(P)$ maximum $dT_{g}(P)/dP<0$, not earlier considered.

## Data Availability

The raw data supporting the conclusions of this article will be made available by the authors on request.

## Abbreviations

The following abbreviations are used in this manuscript:

SG	Simon–Glatzel Equation
C-C	Clausius–Clapeyron Equation
RDK	Rein–Demus–Kechin Equation
LC	Liquid Crystalline Material
BDS	Broadband Dielectric Spectroscopy
DSE	Debye–Stokes–Einstein relation
I-LC	Isotropic Liquid–Liquid Crystal Transition
*P-T*	Pressure–Temperature
